# Hippocampal maintenance after a 12-month physical activity intervention in older adults: The REACT MRI study

**DOI:** 10.1016/j.nicl.2021.102762

**Published:** 2021-07-13

**Authors:** Naiara Demnitz, Afroditi Stathi, Janet Withall, Candida Stainer, Poppy Seager, Jolanthe De Koning, Patrick Esser, Thomas Wassenaar, Helen Dawes, Jonathan Brooks, Klaus P. Ebmeier, Heidi Johansen-Berg, Claire E. Sexton

**Affiliations:** a Department of Psychiatry, University of Oxford, Warneford Hospital, Oxford OX3 7JX, UK; b Danish Research Centre for Magnetic Resonance, Centre for Functional and Diagnostic Imaging and Research, Copenhagen University Hospital – Amager and Hvidovre, 2650 Hvidovre, Denmark; c School of Sport, Exercise and Rehabilitation Sciences, University of Birmingham, Birmingham B15 2TT, UK; d Department for Health, University of Bath, Bath BA2 7AY, UK; e Wellcome Centre for Integrative Neuroimaging, FMRIB Centre, University of Oxford, John Radcliffe Hospital, Oxford OX3 9DU, UK; f Centre for Movement, Occupation and Rehabilitation Sciences, Faculty of Health and Life Sciences, Oxford Brookes University, Oxford OX3 0FL, UK; g Clinical Research and Imaging Centre, University of Bristol, Bristol BS2 8DX, UK

**Keywords:** Exercise, Ageing, RCT, Mobility, Hippocampus

## Abstract

**Background:**

Physical activity interventions have had varying results on modifying hippocampal volume.

**Methods:**

The Retirement in Action (REACT) study conducted a randomised-controlled trial of a 12-month physical activity and behaviour maintenance intervention in older adults at risk of mobility impairments. The physical activity sessions were delivered twice weekly for the first twelve weeks, and then reduced to once weekly, to groups of 15 participants. Activities included cardiovascular, strength, balance and flexibility exercises. A sub-sample of participants in the physical activity (N = 54) and control arms (N = 48) underwent a 3 T MRI brain scan and cognitive assessments at baseline, 6- and 12-months (mean age = 76.6 years, 6.8 SD). It was hypothesised that the intervention would lead to a reduced rate of decline in hippocampal volume. Group differences in changes in cognition were also examined.

**Results:**

As hypothesised, we found a maintenance in left hippocampal volume in the intervention arm, in comparison with the control arm after 12 months (*p* = 0.027). In a secondary analysis, this effect was attenuated after including age, sex and education level as covariates (*p* = 0.057). There was no significant between-group difference in the right hippocampus (*p* = 0.405). Contrary to our hypothesis, we did not find a beneficial effect of the intervention on cognitive outcomes.

**Conclusions:**

Our findings suggest that a community-based physical activity intervention can significantly ward-off hippocampal atrophy in older adults. While the lack of effects on cognition may limit the interpretability of our results, our findings of hippocampal maintenance are promising given the potential clinical relevance of protecting the hippocampus from age-related decline.

## Highlights

• Older adults with poor mobility participated in a 12-month RCT of physical activity.
• 12 months of physical activity facilitates maintenance of left hippocampal volume.
• There was no effect on left or right hippocampal volume after 6 months of training.
• We did not observe a beneficial effect of the intervention on cognitive outcomes.
• Community-based exercise interventions can help promote healthy brain ageing.

## 1 Introduction

The most effective way to improve mobility is through physical activity. Physical activity increases muscle strength, bone density, aerobic capacity and coordination, all well-established determinants of physical function. (McPhee et al., 2016) In addition, physical activity also benefits mobility via an indirect route, as it decreases the risk of chronic diseases (e.g. diabetes, cardiovascular disease, obesity and osteoporosis) which have a detrimental effect on mobility. (Pedersen and Saltin, 2015) Promisingly, randomized controlled trials (RCT) in older adults have shown that mobility outcomes can be effectively modified by physical activity interventions. One such RCT, the Lifestyle Interventions and Independence for Elders study (LIFE, http://clinicaltrials.gov/ct2/show/NCT01072500), tested the effects of a physical activity intervention in sedentary older adults in the United States. As predicted, over 2.6 years of follow-up, this intervention significantly reduced the incidence of major mobility disability. (Pahor et al., 2014)

Physical activity has also been shown to relate to cognitive and brain imaging outcomes. In older adults without known mobility problems, epidemiological studies have shown that individuals who are more physically active have better cognitive function, larger hippocampal volumes and a reduced incidence of dementia. (Blondell et al., 2014, Erickson et al., 2012) Since physical activity is a modifiable behaviour that can be easily targeted, this evidence has spurred the hypothesis that physical activity interventions may reduce the rate of cognitive decline and brain atrophy in older adults. However, RCTs testing this hypothesis have produced inconsistent results – both in cognition (Duzel et al., 2016, Firth et al., 2018) and in brain structure. (Rosano et al., 2017, Niemann et al., 2014, Jonasson et al., 2016, Maass et al., 2015, Burzynska et al., 2017) While several meta-analyses have found that exercise interventions resulted in cognitive improvements across multiple cognitive domains (Colcombe and Kramer, 2003, Smith et al., 2010, Barha et al., 2017, Northey et al., 2018), others have concluded that there is no evidence of exercise-driven cognitive benefits in healthy older adults. (Young et al., 2015) This inconsistency may be due to relatively small sample sizes, the short duration of interventions, or differences between cohorts in physical and cognitive health.

In older adults with mobility difficulties, the evidence is much scarcer and equally inconclusive. A small sub-sample (N = 26) of the LIFE study participants underwent MRI brain scans at baseline and 24-month follow-up. (Rosano et al., 2017) Promisingly, after adjusting for sessions attended and baseline volumes, between-group differences indicated that the physical activity group had significantly larger left hippocampal volumes than the active control group. Albeit only a small sub-sample, these results were indicative of a hippocampal response to a long-term program of moderate-intensity physical activity in adults at risk of developing a mobility impairment.

Driven by the effectiveness of the LIFE trial, the Retirement in Action (REACT) study aimed to conduct a community-based physical activity intervention in older adults in the United Kingdom. Being an efficacy trial, LIFE was highly resource intensive. In contrast, the REACT trial tested the effectiveness of a more pragmatic, low-cost and easily scalable programme. The REACT study randomised participants into two groups: a physical activity and behaviour maintenance intervention arm or a control arm. In order to extend the findings from the LIFE MRI sub-study, a sub-sample of the REACT cohort also underwent detailed cognitive assessments and brain MRI scans at baseline, 6-months and 12-months.

In this paper, we report the pre-specified analysis regarding the cognitive and MRI outcomes of the REACT MRI sub-study. (Stathi et al., 2018) We tested the hypothesis that, compared to control arm participants, participants in the intervention arm would have a reduced rate of decline in hippocampal volume. As a secondary aim, we tested the hypothesis that the intervention would also lead to a reduced rate of cognitive decline in comparison to the control group.

## 2 Methods

### 2.1 Overview of the parent study

The REACT study is a multi-centre, two-arm, single-blinded RCT aimed at evaluating the effectiveness of a community group-based physical activity intervention for preventing decline in mobility (trial registration ID: ISRCTN45627165). The REACT study recruited 777 adults aged 65 years or over who were at risk of developing mobility impairments (defined as scoring between 4 and 9 on the Short Physical Performance Battery, SPPB, Guralnik et al., 1994). Exclusion criteria included: having a neurodegenerative disorder, any terminal illness, severe arthritis significantly restricting mobility, unstable heart conditions (e.g. uncontrolled arrhythmia or angina), severe lung or kidney diseases, implanted cardiac defibrillator, currently receiving radiation therapy or chemotherapy treatment for cancer, awaiting knee or hip surgery, major heart surgery or spinal surgery in the last 6 months and any other clinical condition that the individual’s physician (General Practitioner) considered would make them unsuitable for participation in a physical activity rehabilitation programme. Potential participants were further screened for eligibility via telephone and face to face meetings. Recruited participants were then randomised to either a physical activity and behavioural maintenance intervention or a control arm. (Withall et al., 2020)

The physical activity (PA) intervention consisted of a 12-month exercise and behaviour maintenance program designed for delivery in community centres by qualified exercise professionals. Exercise sessions were organised as group activities with up to 15 participants per group and included walking, functional strength, balance and flexibility exercises aimed at improving lower extremity-function. Sessions began with a progressive warm-up, followed by one of three exercise sets: (a) bilateral lower limb focus with a supplementary lower limb task, (b) unilateral lower limb focus with a supplementary upper limb task or (c) tasks focusing on balance and rotational control. Game-based activities with an aerobic conditioning component were incorporated toward the end of each 60-minute session. These included additional physical challenges, such a hand-eye coordination and balance*.* A third of the PA classes were followed by a 20-minute social session, which encouraged a ‘social club’ atmosphere, provided PA and health information and promoted long-term compliance. In the first 12 weeks of the PA intervention, participants attended two PA sessions per week. After week 12, the exercise session frequency was reduced to one weekly session. Participants allocated to the control arm were invited to 3 60-minute group sessions over the duration of the study. These sessions consisted of talks by invited lecturers on healthy ageing (e.g. nutrition and sleep, but not physical activity).

### 2.2 Participants

**Fig. 1:**
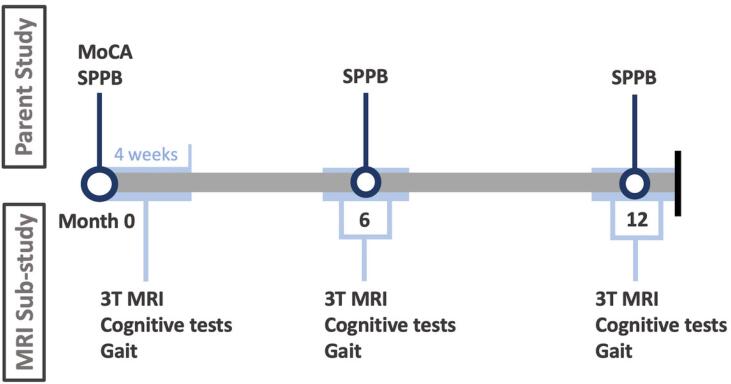
Timeline of data collection. The Short Physical Performance Battery (SPPB) was completed at the assessment days in the parent study, at baseline and after 6 and 12 months. The Montreal Cognitive Assessment (MoCA) was collected at baseline in the parent study. At the MRI sub-study assessment days, which occurred within 4 weeks of the parent study assessment days, participants underwent a 3 T brain MRI, and completed cognitive and gait assessments. The parent study included additional measures and a further time-point (24 months), but only the measures and time-points included in the MRI sub-study are depicted here.

REACT participants were informed about the MRI sub-study at baseline assessment and, if interested in taking part, provided their contact details and consented to being contacted by a member of the MRI research team. On a first-come-first-served basis, interested REACT participants were telephoned, provided with further information about the MRI sub-study and screened for MRI-safety. Sample size balance between groups was ensured by an unblinded researcher. Participation in the MRI sub-study consisted of 3 visits to the Clinical Research and Imaging Centre (CRICBristol) at the University of Bristol. Each visit was scheduled to occur within 4 weeks of the parent study assessment day - at baseline, 6-months and 12-months (Fig. 1).

Eligible participants had no history of psychiatric or neurological illness and had no contraindication for an MRI scan (e.g. pacemaker, metal implant or claustrophobia). On the first visit, participants were briefed, given a chance to ask questions and once again screened for MRI safety, before providing written consent. Ethical approval was obtained from the National Health Service South East Coast-Surrey Research Ethics Committee (REC15/LO/2082).

### 2.3 Hippocampal volumes

MRI data were acquired at CRICBristol on a 3 T Skyra MRI scanner (Magnetom, Siemens, Germany) using a 32-channel head coil. The MRI protocol was adapted from the UK Biobank MRI study. (Miller et al., 2016) T1-weighted images were acquired using a 3D MPRAGE sequence in the sagittal orientation (voxel size = 1x1x1mm, TR = 2000 ms, TE = 2.03 ms, FOV = 208x256x256mm, acquisition time = 4m54s).

MRI data were pre-processed using tools from the FMRIB Software Library (FSL; Woolrich et al., 2009) embedded in the UK Biobank MRI processing pipeline. (Alfaro-Almagro et al., 2018) The pipeline applies a gradient distortion correction to the T1 image, cuts down the field of view to reduce the amount of non-brain tissue, calculates a linear and then non-linear registration to the standard atlas (MNI152, nonlinear 6th generation), applies brain extraction using BET (Smith, 2002) and defaces the image.

Hippocampal volumes (mm^3^) were derived from the pre-processed T1 images using FIRST (Patenaude et al., 2011). All volumes were visually inspected and manually edited when necessary. To ensure intra-rater reliability amongst manual corrections, 10 corrections of FIRST hippocampal segmentations were repeated after 1 month. The intra-class correlation coefficient (ICC = 0.98, 95% CI: 0.90–0.99) indicated consistency between measures. Manual corrections were performed blinded to group allocation to avoid experimenter bias. Hippocampal volumes were adjusted for head size using the overall volumetric head-size scaling factor from SIENAX. (Alfaro-Almagro et al., 2018)

### 2.4 Cognitive measures

Participants in the REACT MRI sub-study completed two measures of executive function, the flanker and the two-back tasks, and one measure of relational memory, the Object Location test.

In the Flanker task, participants were presented with five large arrows in a row in the centre of the screen. In congruent trials, all arrows pointed in the same direction. In incongruent trials, the direction of the middle arrow (target) was the opposite of the other arrows. Participants were instructed to indicate the direction of the target arrow by pressing the “F” or “J” key of a keyboard when the arrow pointed left or right, respectively. The task consisted of a practice block and 2 test blocks with 52 trials each. The interference effect was calculated as:

$$\left(\frac{\mathrm{meanRTofcorrectincongruentresponses}-meanRTofcorrectcongruentresponses}{\mathrm{meanRTofcorrectcongruentresponses}}\right)\times 100$$

In the Two-Back task, participants were shown a sequence of visual stimuli, one at a time, and instructed to indicate, via key press, whether the current stimulus was identical to the one presented before-last or not. The visual stimuli consisted of clip-art images of objects, such as a star, rainbow or spiderweb. The task consisted of two practice blocks and two test blocks. Accuracy scores represent the percentage of correct responses out of the total number of trials (48). Reaction time (ms) was computed as the average latency of correct responses. The two-back and flanker tasks were administered using Millisecond software (Inquisit 4 Lab, 2016).

The object-location paradigm, described in detail elsewhere, required participants to remember the identity and location of novel objects. (Pertzov et al., 2015) After the initial presentation of three items and a subsequent delay of one or four seconds, participants were instructed to identify the object they had previously seen and then drag it to its remembered location on the touchscreen computer monitor. Object identity was computed as a percentage of fractals correctly identified in the test block. To calculate localisation memory, the distance between the centre of the location of the participant’s response and the centre of the correct object location in the paradigm was computed. To quantify misbinding errors, the rate at which a target was identified correctly, but placed within a 4.5˚ radius of one of the non-target locations, was calculated. The object-location task was administered using the Cogent toolbox for Matlab (v2016a, Mathworks) on a touchscreen computer (DELL 9030 All-in-One, 23 in.).

The Montreal Cognitive Assessment (MoCA) was used as a measure of global cognition. With a maximum score of 30, scores below 26 are suggestive of cognitive impairment. (Nasreddine et al., 2005) The British Columbia Cognitive Complaints Inventory (BC-CCI), a 6-item scale, was used to measure perceived cognitive problems. (Iverson and Lam, 2013) With a maximum score of 18, scores above 5 are indicative of cognitive complaints.

### 2.5 Mobility measures

The Short Physical Performance Battery (SPPB ^19^) was administered at the parent REACT assessment days. The SPPB measures functional performance through 3 tests: 4-m gait speed, standing balance, and timed measure of 5 repeated sit-to-stands. The performance on the 3 tasks is combined into one score between 0 (poor mobility) and 12 (good mobility).

At the REACT MRI sub-study assessments, participants completed two walks along a 10-meter course in a corridor. Participants were instructed to walk at their own speed and without the use of walking-aids. An inertial measurement unit (MTX, Xsens, Netherlands) was used to calculate spatio-temporal gait parameters. For a description of each gait measure, please see Auvinet et al., 2002, Esser et al., 2013, Appendix A.

### 2.6 Sample characteristics

Body mass index was measured based on weight in kilograms divided by height in square meters. Education level was collected through self-report and ranged from 1 to 7, depending on highest education level. Education levels corresponded to: (1) primary school, (2) middle school, (3) some secondary school, (4) completed secondary school, (5) some college or vocational training, (6) completed college or university, or (7) completed graduate degree, or higher.

### 2.7 Statistical analyses

Statistical analyses were pre-specified in the analysis plan published within the REACT protocol (Stathi et al., 2018) and performed by a researcher blinded to treatment status.

Analyses followed a complete-case approach, in which all participants with available outcomes were included, regardless of their adherence to the intervention. Each outcome (change between 12-months and baseline) was first examined using a one-way analysis of variance (ANOVA) with group (intervention, control) as a between-subjects factor. If an effect was found to be significant, an analysis of covariance (ANCOVA) model was carried out to include age, sex and education as covariates. In addition, when significant effects were identified, single-sample t-tests were conducted to assess whether the change differed from zero. Homogeneity of variance was verified using the Levene test.

Sub-group comparisons, specified a-priori in the study protocol (Stathi et al., 2018), were conducted for sex, baseline MoCA scores (≤26 vs > 26), median age, and adherence to intervention (high vs low). These were assessed using linear regressions, with the interaction between group and strata (e.g., sex) as the independent variable and change in the outcome measure (e.g., hippocampal volume) as the dependent variable.

Partial correlations were calculated to examine the relationship between change after 12-months in hippocampal volume and cognitive outcomes in the intervention group. Change-change correlations were also computed for the relationship between SPPB, MRI and cognitive outcomes. All correlations included age, sex and education level as covariates. For our primary analyses on hippocampal volumes, the alpha level was set at *p* < 0.05. For secondary cognitive analyses, the alpha level was Bonferroni-corrected for 7 cognitive outcomes and set at *p* < 0.007.

All analyses were conducted using the Statistical and Machine Learning Toolbox (v10.2) for MATLAB (R2016a; MathWorks). Effect sizes (omega squared) were calculated using the MES Toolbox in MATLAB (Hentschke and Stuttgen, 2011) and plots were generated using the ggplot2 (Wickham and Sievert, 2016) and ggstatsplot packages (Patil, 2018) in R version 3.5.1.

## 3 Results

**Fig. 2:**
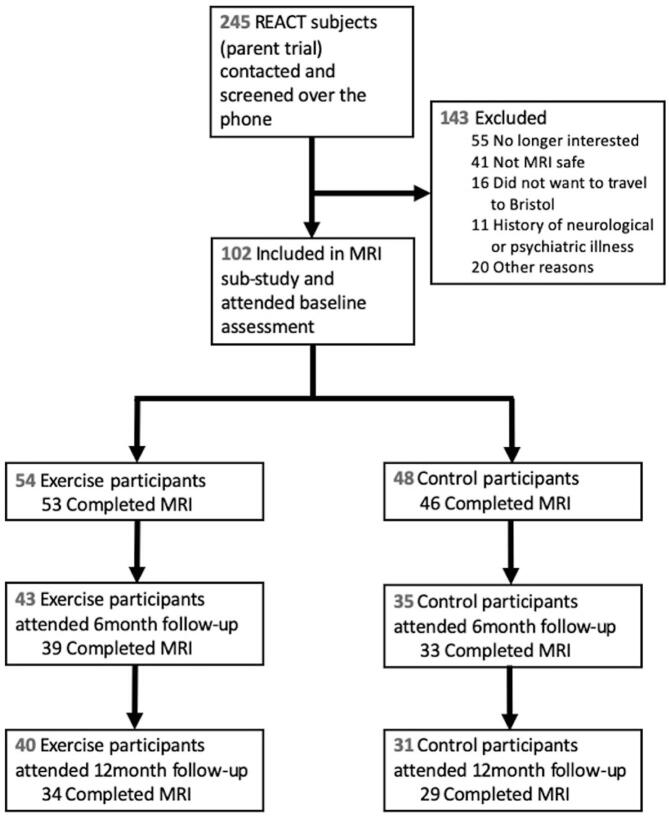
Flow diagram of participant attrition.

One hundred and two participants completed baseline assessments and were included in the REACT MRI sub-study (Table 1, Fig. 2). The sample had an average age of 76.56 years (SD 6.78), had a larger proportion of females (62%), an average BMI categorised as ‘overweight’ (mean = 28.86, SD = 5.23), and the majority had completed at least ‘some college or vocational training’ (53%). On average, participants had a higher level of education (4.75 vs. 2.79, p < 0.001) and a higher MOCA score at baseline (25.11 vs. 24.37, p = 0.04) than participants in the parent cohort (Appendix B). Other baseline characteristics, such as age, SPPB score and sex, were similar to those of the parent study. Complete MRI datasets for baseline and 12-month follow-up were available for 34 intervention and 29 control participants. In terms of cognitive measures, 40 and 31 complete datasets were available from intervention and control groups, respectively. Mean adherence to the intervention, as a percentage of exercise classes attended, was 65.2% (SD = 27.4).

**Table 1:** Baseline characteristics of each group.

	**Physical activity group**		**Control group**	
	**Mean (SD)**	**Range**	**Mean (SD)**	**Range**
Sample characteristics (n)	*54*		*48*	
Age	76.12 (6.81)	65.62 – 88.17	77.05 (6.78)	66.53 – 92.88
Female (N, %)	33 (61.1%)		30 (62.5%)	
Education level	5 (1.01)	3 – 7	4.56 (0.79)	3 – 6
MoCA	25.54 (3.03)	17 – 30	24.65 (3.70)	16 – 30
SPPB	7.67 (1.58)	4 – 9	7.51 (1.29)	4 – 9
BMI	29.39 (5.68)	20.4 – 43.95	28.26 (4.66)	20.99 – 42.17
BC-CCI (total score)	6.11 (4)	0 – 14	7.15 (3.89)	0 – 14
Walking speed (m/s)	1.16 (0.21)	0.7 – 1.56	1.14 (0.20)	0.66 – 1.58
MRI measures (n)	53		46	
Left Hippocampal volume	4071.5 (743.4)	1479.8 – 5763.5	4140.4 (506.1)	2953.4 – 5201.3
Right Hippocampal volume	4327.3 (723.5)	1626.1 – 5573.7	4433.6 (512.5)	3461.3 – 5642.8
Cognitive measures (n)	54		48	
Object Location – Identity (% correct)	87.9 (6.66)	64 – 98	86.29 (5.68)	74 – 94
Object Location – Localisation memory (error)	8.05 (2.39)	4.86 – 16.61	9.55 (2.48)	4.72 – 15.03
Object Location – Misbinding error	0.14 (0.08)	0.02 – 0.35	0.19 (0.09)	0.02 – 0.35
Flanker interference score^a^	12.83 (8.44)	−21.69 – 40.67	13.72 (8.08)	−11.97 – 28.91
Two-back mean RT (ms)	1252.2 (181.32)	887.69 – 1569.1	1237.3 (206.91)	444.33 – 1512.9
Two-back mean accuracy	29.75 (8.07)	9 – 46	25.17 (9.2)	3 – 39

Note. ^a^ One participant did not complete the Flanker task.

### 3.1 Group differences at 12 months

Outcome measures (change from baseline to 12-months) were compared between-groups using ANOVAs. All comparisons satisfied Levene’s test for equality of variances (Table 2).

**Table 2:** Group differences in change between intervention and control participants at 12 months. Mean (SD) of change are presented for each group.

	**Physical activity group**	**Control group**	**Group difference in change**		
			***F***	***p***	*ω*^2^
***Change in MRI measures (n)***	*34*	*29*			
Right hippocampal volume (mm^3^)	−192.69 (257.34)	−240.63 (183.27)	0.70	0.405	−0.005
Left hippocampal volume (mm^3^)	**−99.96 (253.47)**	**−236.34 (241.86)**	**5.12**	**0.027**	**0.061**
					
***Cognitive measures***	*40*	*30*			
Two-Back Accuracy	**0.39 (7.71)**	**6.70 (9.62)**	**8.6**	**0.005**	**0.105**
Two-Back Reaction Time (ms)	−1.11 (162.2)	−21.53 (172.65)	0.24	0.628	−0.012
Flanker (interference)	−0.47 (11.01)	1.01 (10.48)	0.33	0.569	−0.01
Object Location: identification accuracy (%)	−21 (38)	−27 (43)	0.7	0.403	0
Object Location: Location errors	−0.34 (2.04)	−0.75 (2.03)	0.68	0.411	−0.005
Object Location: Misbinding error	0.03 (0.13)	<0.01 (0.13)	1.24	0.27	0.033
Subjective cognitive complaints (BC-CCI)	−0.25 (3.76)	−1 (4.06)	0.64	0.428	−0.005

Significant between-group differences are highlighted in bold.

**Fig. 3:**
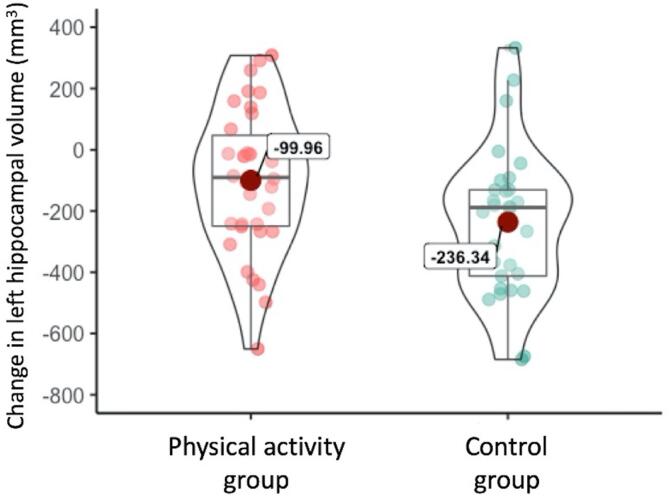
Hippocampal volume decreased in both groups over 12 months, but this decrease was significantly greater in the control group. Violin plots of mean change illustrate the difference in change (12 months – baseline) in left hippocampal volume between groups. The outer edge of each violin plot depicts the distribution of data values along the y-axis (density trace), while the inner hour-glass shape indicates the interquartile range.

Analyses of hippocampal volume change revealed a significant difference between groups for the left (F(1,61) = 5.12, p = 0.027, *ω*^2^ = 0.061), but not the right, hippocampus. The group difference for left hippocampus reflected greater reduction in volume in the control group compared to the intervention group. Both groups showed significant decline in left hippocampal volume over time (single sample *t*-test compared to zero: t(33)_Physical activity_ = -2.48, p = 0.019; t(28)_Control_ = -5.26, p < 0.001; Fig. 3).

Analysis of change in cognitive measures revealed a significant difference between groups for accuracy in the two-back task (*F*(1,63) = 8.6, *p* = 0.005, *ω*^2^ = 0.105). Participants in the control group improved their accuracy, and this increase in score significantly differed from zero (t(26) = 3.62, p = 0.002), while change in the intervention group did not (t(37) = 0.32, p = 0.754). No other cognitive measures showed significant between-group differences.

There was a significant group difference for change in SPPB scores (F(1,84) = 10.27, p = 0.002, *ω*^2^ = 0.097). While SPPB scores increased in the intervention group (t(46) = 4.9, p < 0.001), change in scores in the control group did not differ from zero (t(38) = 1.72, p = 0.461). No significant group differences in change were observed for any gait outcome (Appendix C).

ANCOVA models, including age, sex and education as covariates, were applied to the outcome measures that emerged as significant in the primary ANOVA analyses. The between-group difference in left hippocampal volume (*F*(1,58) = 3.77, p = 0.057, *ω*^2^ = 0.042) and two-back accuracy (*F*(1,60) = 5.55, p = 0.022, *ω*^2^ = 0.062; Bonferroni-corrected threshold p > 0.007) were no longer significant after accounting for covariates. The between-group difference in SPPB (*F*(1,81) = 13.15, p < 0.001, *ω*^2^ = 0.118) remained significant after accounting for covariates.

### 3.2 Group differences at 6 months

There was no significant difference in change in hippocampal volume between groups, in either hemisphere, after 6 months. The between-group difference in change in localisation errors was not significant after Bonferroni-correction (*F*(1,74) = 5.45, *p* = 0.022, *ω*^2^ = 0.06; Bonferroni-threshold *p* > 0.007). There were no further between-group differences in cognitive outcomes (Appendix D).

### 3.3 Sub-group analyses

Linear regressions with interaction terms were conducted for pre-specified sub-group analyses of 12-month effects. Intervention effects on MRI outcomes did not vary by sex, baseline MoCA, or adherence to intervention (Appendix E). An interaction was observed with sex (p = 0.009) and age group (p = 0.026) on the two-back test, but no other cognitive measure.

ANOVA analyses on 12-month MRI and cognitive outcomes were repeated in the stratified sub-groups, and these are reported in the appendices for descriptive purposes only.

### 3.4 Change-change correlations

Correlations between change over 12-months in left hippocampal volume and cognition were computed for participants in the intervention group. After adjusting for age, sex and education level, change in left hippocampal volume was not associated with change in any cognitive outcome (Appendix F).

In an exploratory analysis, we tested whether change in SPPB over 12-months correlated with change in hippocampal volume or cognitive outcomes in the intervention group. With age, sex and education level as covariates, change in SPPB was positively correlated with change in accuracy on the two-back task (r = 0.45, p = 0.008), and negatively correlated with subjective cognitive complaints (r = -0.37, p = 0.03). No correlation survived corrections for multiple comparisons (Appendix G).

## 4 Discussion

This study aimed to test the hypothesis that a 12-month physical activity and behavioural maintenance intervention resulted in beneficial effects on hippocampal volume and cognition in a sample of older adults at risk of mobility impairments. In line with our prediction, we found a greater decrease in left hippocampal volume in the control arm, suggesting reduced decline in hippocampal volume in the intervention group. It is important to note, however, that when covarying for education, sex and age, the effect of the intervention on the left hippocampal volume became non-significant. Further, we found no evidence to support our hypothesis that the PA intervention elicited beneficial effects on cognitive outcomes.

### 4.1 MRI outcomes

There is considerable evidence from observational studies to suggest a correlation between physical activity and the volume of the hippocampus (Erickson et al., 2012, Erickson et al., 2009, Hamer et al., 2018), a region crucial for memory and implicated in Alzheimer’s disease. (Van Petten, 2004) However, evidence for the effect of physical activity RCTs on hippocampal volume has been inconsistent (Rosano et al., 2017, Niemann et al., 2014, Jonasson et al., 2016, Maass et al., 2015, Burzynska et al., 2017). Pooling across RCTs, two meta-analyses have found a significant effect of exercise on bilateral (Wilckens et al., 2021) and left (Firth et al., 2018) hippocampal volumes. Both meta-analyses indicate that these effects were driven by a maintenance, rather than an increase, of hippocampal volume in the exercise conditions. Accordingly, our findings show less decline in left hippocampal volume after 12-months of a physical activity intervention.

Our findings are also in line with those reported by the LIFE MRI sub-study. (Rosano et al., 2017) This is despite several notable differences between the two studies - the LIFE MRI sub-study was much smaller (N = 26), utilised a higher magnetic field strength (7T) and had a longer duration (24-months). The length of the follow-up appears to be particularly important. In the systematic review by Firth and colleagues (2018), the significant effects of exercise on hippocampal volume only emerged from interventions of 12-months or longer. (Rosano et al., 2017, Erickson et al., 2011) This pattern of findings has raised the possibility that longer interventions are necessary to observe exercise-driven effects in older adults. In contrast, a more recent meta-analyses found a significant overall effect in studies 6 months or longer. (Wilckens et al., 2021) In our own analyses, the effect on hippocampal volume was observed after 12, but not 6, months.

There was no interaction between the effect of the intervention and sex, age group or baseline MoCA scores on hippocampal volume in our sub-group analyses. Nonetheless, the patterns observed in exploratory sub-group analyses may serve to generate hypothesis regarding which groups are most susceptible to the effects of physical activity (Appendix E). In analyses stratified by age, a significant difference in change was noted in left hippocampal volume in the younger, but not older, sub-group. Similarly, a significant difference in left hippocampal volume was observed in females, but not in males, and in those with higher (≥26; i.e. fewer cognitive impairments), but not lower, baseline MOCA scores. While the observed sex differences are in line with the hypothesis that the effects of exercise are most evident in women (Kramer and Erickson, 2007), our MOCA sub-group analyses contribute to a murky body of literature. A previous 6-month RCT of PA in women with mild cognitive impairments found increased hippocampal volume in the intervention group (ten Brinke et al., 2015), but others have found no beneficial effects of a PA intervention on hippocampal volume in adults at risk of Alzheimer’s Disease. (Venkatraman et al., 2020) Altogether, our results suggest that the hippocampi of younger, female, and cognitively healthier adults are most likely to benefit from physical activity. While highlighting these trends might be an interesting hypothesis-generating exercise, it is important to not over-interpret these findings given the small sample sizes of the sub-groups. For instance, as there were more females than males in our sample, it could be that our male sub-group was simply underpowered to identify group differences in hippocampal volume.

### 4.2 Cognitive outcomes

Except for accuracy on the two-back task, no differences in change were observed between groups in objective or subjective cognitive measures. These findings add to a mixed body of literature.

Several systematic reviews and meta-analyses have aimed to clarify the effects of physical activity on cognition, yet even across reviews the results have proved inconsistent. Two meta-analyses have previously observed that physical activity interventions improved attention, memory, executive function and processing speed in older adults. (Colcombe and Kramer, 2003, Smith et al., 2010) In contrast, a Cochrane review of 12 physical activity RCTs in adults aged over 55 found no evidence of a cognitive benefit from aerobic exercise in any cognitive domain. (Young et al., 2015) In agreement with the most recent reviews, we did not observe any exercise-driven cognitive improvements in our intervention.

Although our results show a difference in change between groups in the two-back task, indicating an improvement in performance in the control group, this finding must be interpreted with caution. Crucially, this cognitive measure already differed at baseline with PA participants having higher accuracy scores than control participants. Accordingly, it is plausible that the poorer performance of control participants on this measure enabled them to show a greater improvement (change) than physical activity participants, who performed fewer errors at the start. In line with this hypothesis, a post-hoc comparison of change in accuracy in the two-back task, this time with baseline scores as a covariate, notably reduced the effect of the intervention on change in accuracy (*F*(1,62) = 4.31, *p =* 0.042, *ω*^2^ = 0.031).

The included tasks targeted cognitive domains that have previously been shown to be most sensitive to changes in physical activity: relational memory and executive function. (Duzel et al., 2016, Sink et al., 2015) Nonetheless, our targeted testing means that changes in other cognitive domains, not assessed here, would not have been captured. A wider range of cognitive domains were assessed in the parent cohort, and yet these also revealed null results (Stathi et al, under review). Further, the cognitive tasks were notably difficult – as evidenced in feedback from participants and by low average scores at baseline. The measures may, therefore, have been too challenging to allow improvements in either group. In reference to our two-back task, it is also worth noting that alternative methods for calculating performance exist, and these may lead to more targeted measures of working memory. For example, comparing 2-back to 1-back performance more rigorously separates working memory capacity from other cognitive and motor processes required. Finally, we cannot dismiss the possibility that the effects of the intervention may take longer to manifest in cognitive tests than in hippocampal volume.

### 4.3 Methodological limitations and strengths

The attrition rate for the primary outcome at 12-months was 39%. In contrast, the attrition rate for the primary outcome in the parent trial at 24 months was 19.2%. The increased attrition rate in the MRI sub-study was partly due to the travel demand of attending the testing sessions. Participants were based across the south-west of England and travelling to the MRI centre in Bristol became an increasing barrier as the study went on. To test the impact of missing data on our primary analysis, we carried out a sensitivity analysis in all participants for whom at least 2 scans are available (76.5% of randomised sample), with imputations using the last available outcome (6-months) carried forward (to 12-months). The effect observed on left hippocampal volume remained with this approach (F(1, 76) = 4.49, 0.037). However, this arguably introduces an additional bias: we are now comparing change in 6-months together with change in 12-months. Since we expect the hippocampus to decline within this timeframe and there were more physical activity participants than control participants carried forward, this approach arguably biases our analyses towards over-estimating the effect of the intervention. On average, intervention group participants in the MRI sub-study attended 65.2% (SD = 27.4) of their physical activity sessions. In the parent trial, intervention group participants attended, on average, 67.7% (SD = 24.9) of their physical activity sessions. Adherence (high vs. low) did not significantly moderate the effect of the intervention on left hippocampal volume (Appendix E). Still, it is important to note that attendance does not necessarily reflect duration or ‘intensity adherence’, in relations to the volume or intensity of engagement during the sessions. (Rivera-Torres et al., 2019).

The role of the hippocampus extends beyond cognition. Preserving hippocampal volume may, for example, have beneficial effects on sleep quality (Fjell et al., 2020) and depressive symptoms. (Videbech and Ravnkilde, 2004) While the current study was not designed to detect these changes, it would be beneficial for future studies to also consider the potential benefits of maintained hippocampal volume in relation to sleep and depressive symptoms.

A central feature of the intervention was its group delivery. The group format of the classes was aimed at promoting adherence and social interaction among participants. Given previous reports of a protective role of social activity on the brains of older adults, (Kelly et al., 2017, Anaturk et al., 2018) our group comparisons cannot isolate the effects from the physical activity and the social activity components of the intervention. Despite being delivered in a group setting, exercise programmes were personalised to each participant, based on their functional status and goals, and using rate of perceived exertion (RPE) methods (a 15-point numerical scale ranging from 6 to 20). (Borg, 1982) During the 12-month exercise intervention, strength-based exercises were prescribed to reflect intensities rated ‘moderate to vigorous’ (11 to 16). Towards the end of each session, games-based activities of 15 to 20 min duration were delivered at ‘light to moderate’ intensities (8 to 13). By accommodating for daily fluctuations in residual muscle soreness or fatigue, RPE methods encourage more tolerable and enjoyable adjustments to individual training loads on a session-by-session basis, (Buskard et al., 2019) an important consideration for the long-term adherence to any exercise intervention for older adults. (Chodzko-Zajko et al., 2009) Therefore, this individualised approach to exercise prescription enabled each participant to progress at their own pace. However, we acknowledge this method also introduced a wide range of inter-individual variability in the intensity of the exercises. Since the study did not measure change in cardiorespiratory fitness, we were unable to explore how this factor is associated with our outcome measures. Further, it is important to note that the control group was not an active one – participants were invited to attend 3 educational sessions for the duration of the study. Between-group effects could therefore also relate to increased motivation and engagement in activity in the active group. While these differences may limit the understanding of the mechanisms underlying the effects of the intervention, the social nature of the intervention sessions was also a notable strength. In addition to being cost-effective, the social activity endorsed by a group delivery design has been found to promote long-term compliance and motivation in previous RCTs. (Farrance et al., 2016)

Besides being pragmatic, community-based, well-powered, and a randomised controlled-trial, REACT had several methodological strengths. In a systematic review of 29 exercise RCTs, less than half utilised blinded assessments. (Smith et al., 2010) Here, assessments and data-analyses were conducted blinded to group allocation. Further, Young and colleagues noted that out of 12 RCTs in older adults, none of the trials had published protocols so it was not possible to tell if there was selective reporting of results. (Young et al., 2015) In contrast, the main analyses presented in this paper were stipulated a priori. (Stathi et al., 2018) Any analyses outside of the published protocol are presented as exploratory.

Other factors, not captured here, might modify the effect of activity programmes on the brain, such as APOE4 status (Obisesan et al., 2012), adherence to a Mediterranean diet (Tangney et al., 2011) and social support. (Stranahan et al., 2006) These factors were not measured in our study, but could be of interest for future interventions as they may help to disentangle the mixed findings in the existing literature.

## 5 Conclusions

This pragmatic 12-month intervention had a positive effect on the left hippocampal volume of older adults at risk of mobility impairments, whereby the physical activity group showed less hippocampal decline. However, the intervention did not significantly improve cognitive performance and changes in hippocampal volume were not associated with cognitive changes. In the absence of cognitive changes, it is difficult to interpret the functional significance of the observed maintenance in hippocampal volume. Further studies are needed to determine whether, and under what conditions, effects of exercise on the hippocampus have longer-term consequences to prevent and delay neurodegeneration.

## Funding

This work was supported by the NIHR Public Health Research programme (13/164/ 51), NIHR Oxford Health Biomedical Research Centre (BRC) and the NIHR Oxford BRC based at Oxford University Hospitals NHS Trust and University of Oxford. HJB is supported by the Wellcome Trust (110027/Z/15/Z). The Wellcome Centre for Integrative Neuroimaging is supported by core funding from the Wellcome Trust (203139/Z/16/Z).

## Conflicts of interest

CES is now a full-time employee of the Alzheimer’s Association.

## CRediT authorship contribution statement

**Naiara Demnitz:** Conceptualization, Formal analysis, Writing - original draft. **Afroditi Stathi:** Conceptualization, Funding acquisition, Project administration. **Janet Withall:** Conceptualization, Project administration. **Candida Stainer:** Investigation, Project administration. **Poppy Seager:** Investigation, Project administration. **Jolanthe De Koning:** Investigation, Project administration. **Patrick Esser:** Resources, Formal analysis. **Thomas Wassenaar:** Validation. **Helen Dawes:** Resources. **Jonathan Brooks:** Methodology. **Klaus P. Ebmeier:** Methodology, Supervision. **Heidi Johansen-Berg:** Conceptualization, Methodology, Resources, Funding acquisition, Supervision. **Claire E. Sexton:** Conceptualization, Methodology, Supervision.

## Declaration of Competing Interest

The authors declare that they have no known competing financial interests or personal relationships that could have appeared to influence the work reported in this paper.
