# Supplementary material for: Hippocampal maintenance after a 12-month physical activity intervention in older adults: The REACT MRI study
Source: Neuroimage Clin. 2021 Jul 13;35:102762. doi: 10.1016/j.nicl.2021.102762 (PMC9421470; doi:10.1016/j.nicl.2021.102762)
Supplement: Supplementary data 1 [file mmc1.docx]

**Supplementary Materials**

**Appendix A. Description of collected spatio-temporal gait measures.**

An inertial measurement unit (MTX, Xsens, Netherlands), measuring at a sample frequency of 100 Hz, was placed over the projected centre of mass (CoM) of each participant, located over the fourth lumbar vertebra. The inertial measurements were calculated according to Zijlstra’s inverted pendulum model (Auvinet et al., 2002; Esser et al., 2013). For each walk, a series of temporal, spatial and variability measures were derived using a program developed using LabVIEW2015 (National Instruments, Ireland).

Walking speed (m/s) refers to the time taken to cover 1metre in the 10-meter path. Stride length (m) indicated the distance between two successive steps of the same foot. Stride time (ms) is the time interval between two consecutive steps of the same foot. The variability within these 3 measures was also computed. For this, the coefficient of variation (CoV), which describes the ratio of the standard deviation (SD) to the mean, was derived - thus leading to 3 additional variables: walking speed CoV, stride length CoV and stride time CoV**.** Values above or below 3 standard deviations from the mean were deemed as outliers and excluded from the analysis.

**Appendix B. Baseline characteristics of participants in the REACT MRI Sub-study and participants in the REACT parent cohort. Values are Mean (SD) or N (%).**

| **Sample characteristic** | **REACT MRI sub-study  (n = 102)** | **REACT parent cohort  (n = 777)** | **Test statistic** | ***P value*** |
| --- | --- | --- | --- | --- |
| Age | 76.56 (6.78) | 77.55 (6.79) | -1.39 | 0.17 |
| Female (N, %) | 63 (61.8%) | 514 (66.2%) | 0.78 | 0.38 |
| Education level | 4.75 (0.95) | 2.79 (1.06) | 19.24 | <0.001 |
| MoCA | 25.11 (3.38) | 24.37 (3.66) | 2.03 | 0.04 |
| SPPB | 7.60 (1.45) | 7.37 (1.56) | 1.41 | 0.16 |

Pearson’s Chi-Squared test was used to compare number of females in the two samples. Two Sample T-Tests were conducted for all other measures.

**Appendix C. There was no main effect of intervention of gait outcomes.**

Group differences in change in measures of gait between exercise and control participants. Mean (SD) of change are presented for each group.

|  | **Physical activity** | **Control group** | **Group difference in change** | | |
| --- | --- | --- | --- | --- | --- |
|  |  |  | ***F*** | ***p*** | ***ω2*** |
| ***Change in gait measures (n)^1^*** | *31* | *27* |  |  |  |
| Walking speed (m/s) | 0.11 (0.16) | 0.06 (0.17) | 1.16 | 0.286 | 0.003 |
| Step time (ms) | -24.68 (33.62) | -10.44 (33.01) | 2.58 | 0.114 | 0.027 |
| Stride length (m) | 0.03 (0.1) | 0.01 (0.1) | 0.509 | 0.478 | -0.021 |
| Walking speed CoV | -0.03 (0.1) | -0.03 (0.04) | 0 | 0.977 | -0.018 |
| Step time CoV | -18.89 (26.67) | -16.99 (15.91) | 0.11 | 0.746 | -0.016 |
| Stride length CoV | -0.02 (0.03) | -0.03 (0.03) | 3.44 | 0.07 | 0.048 |

^1^Due to a technical fault, baseline gait measures were not obtained from 14 participants. In addition, the gait from one participant was deemed as an outlier (-3 SDs from the mean) and excluded.

**Appendix D. Effects of intervention after 6 months.**

Group differences in change between exercise and control participants. Mean (SD) of change are presented for each group.

|  | **Physical activity** | **Control group** | **Group difference in change** | | |
| --- | --- | --- | --- | --- | --- |
|  |  |  | ***F*** | ***p*** | ***ω2*** |
| ***Change in MRI measures (n)*** | *39* | *33* |  |  |  |
| Right hippocampal volume (mm^3^) | -46.19 (196.92) | -39.63 (202.91) | 0.02 | 0.889 | -0.014 |
| Left hippocampal volume (mm^3^) | 3.66 (161.61) | -23.31 (215.41) | 0.37 | 0.546 | -0.009 |
|  |  |  |  |  |  |
| ***Change in cognitive measures (n)*** | *43* | *35* |  |  |  |
| 2Back Accuracy | 0.80 (7.19) | 1.84 (9.46) | 0.28 | 0.601 | -0.011 |
| 2Back Reaction Time | -49.08 (205.40) | -12.48 (137.72) | 0.71 | 0.404 | -0.004 |
| Flanker (interference) | -0.15 (8.05) | -0.72 (8.47) | 0.09 | 0.768 | -0.013 |
| Object Location: identification accuracy (%) | 0 (0.05) | 0.01 (0.05) | 0.42 | 0.518 | -0.008 |
| Object Location: Location errors | **0.27 (2.28)** | **-0.88 (1.97)** | **5.45** | **0.022** | **0.055** |
| Object Location: Misbinding error | -0.01 (0.11) | -0.05 (0.13) | 2.73 | 0.103 | 0.022 |
| Subjective cognitive complaints (BC-CCI) | -0.42 (3.71) | -0.69 (3.66) | 0.1 | 0.751 | -0.012 |

**Appendix E. Sub-group analyses.**

**Median Age.**

Sub-group analyses split by age separately considered younger (<76 years, 52%, n=37) and older (≥76 years, 48%, n=34) subgroups. Within the younger age sub-group, there was a significant group difference for change in left hippocampal volume (*F*(1,32) = 7.01, *p*= 0.013, *ω*^2^ =0.15). While the reduction in volume observed in the control group differed from zero (t(14) = -4.96, p <0.001), the change in the intervention group did not (t(18) = -1.29, p = 0.214). Change in accuracy in the two-back task differed between the younger intervention sub-group (*F*(1,34)=13.99, *p*<0.001, *ω*^2^ = 0.265). While mean change in accuracy did not differ from zero for participants in intervention group (*t*(18) = -1.29, *p* = 0.214), there was a significant increase in scores in the control group (*t*(14) = -4.96, *p* < 0.001). In the older sub-group, there were no significant differences in change between control and physical activity groups.

Group differences between exercise and control groups, for each median-split sub-group (aged <76 or 76+ years). Mean (SD) of change are presented for each sub-group.

|  | **Younger adults (<76 years)** | | | | | **Older adults (76+ years)** | | | | |  |
| --- | --- | --- | --- | --- | --- | --- | --- | --- | --- | --- | --- |
|  | **Physical activity** | **Control group** | ***F*** | ***p*** | ***ω2*** | **Physical activity** | **Control group** | ***F*** | ***p*** | ***ω2*** | **P value for interaction** |
| ***Change in MRI measures (n)*** | *19* | *15* |  |  |  | *15* | *14* |  |  |  |  |
| Right hippocampal volume (mm^3^) | -205.21 (290.47) | -279.4 (146.45) | 0.81 | 0.374 | -0.006 | -176.83 (217.13) | -199.08 (213.67) | 0.08 | 0.783 | -0.033 | 0.655 |
| Left hippocampal volume (mm^3^) | **-70.46 (238.46)** | **-281.2 (219.74)** | **7.01** | **0.013** | **0.15** | -137.32 (234.32) | -188.28 (263.03) | 0.3 | 0.586 | -0.025 | 0.193 |
| Global GM volume (mm^3^) | -3357 (17099) | -18752 (15320) | 7.43 | 0.010 | 0.159 | 1903 (17808) | -4485 (12572) | 1.22 | 0.278 | 0.008 | 0.271 |
| Global WM volume (mm^3^) | -865.06 (29115) | 18175 (23296) | 4.25 | 0.047 | 0.087 | -9672 (27621) | 1818 (23742) | 1.43 | 0.242 | 0.015 | 0.574 |
|  |  |  |  |  |  |  |  |  |  |  |  |
| ***Change in cognitive measures (n)*** | *22* | *15* |  |  |  | *18* | *16* |  |  |  |  |
| Two-Back Accuracy^1^ | **-0.57 (7.92)** | **10 (8.94)** | **13.99** | **<0.001** | **0.265** | 1.59 (7.49) | 2.58 (9.13) | 0.104 | 0.75 | -0.032 | **0.026** |
| Two-Back Reaction Time (ms) | 17.12 (114.09) | -85.1 (162.9) | 4.92 | 0.033 | 0.098 | -23.63 (208.8) | 57.93 (155.83) | 1.31 | 0.263 | 0.011 | **0.029** |
| Flanker (interference)^2^ | 1.36 (8.58) | 2.67 (11.92) | 0.15 | 0.699 | -0.024 | -2.84 (13.44) | -0.54 (9.04) | 0.33 | 0.569 | -0.021 | 0.848 |
| Object Location: identification accuracy (%) | 1.41 (4.64) | 2.97 (4.52) | 1.03 | 0.32 | 0 | -0.01 (0.06) | 0.03 (0.04) | 2.84 | 0.102 | 0.051 | 0.511 |
| Object Location: Location errors | 0.01 (0.05) | 0.03 (0.05) | 1.03 | 0.318 | -0.026 | -0.006 (0.06) | 0.026 (0.04) | 2.84 | 0.102 | 0.012 | 0.568 |
| Object Location: Misbinding error | 0.02 (0.12) | 0.01 (0.11) | 0.04 | 0.834 | -0.027 | 0.05 (0.13) | -0.01 (0.15) | 1.69 | 0.202 | 0.02 | 0.380 |
| Subjective cognitive complaints (BC-CCI)^3^ | 0.23 (4.19) | -2.13 (3.87) | 3.01 | 0.09 | 0.052 | -0.83 (3.19) | 0.13 (4.05) | 0.59 | 0.449 | -0.013 | 0.079 |

^1^ Younger: PA_n_ = 21, CG_n_ = 15. Older: PA_n_ = 17, CG_n_ = 12.
^2^ Older: PA_n_ = 17, CG_n_ = 16.

^3^ Older: PA_n_ = 18, CG_n_ = 15.

**Baseline MOCA scores**Sub-group analyses split by MOCA score separately considered those with higher (≥26, 55%, n=39) and lower (<26, 45%, n=32) scores at baseline. In those with higher MOCA at baseline, there was a significant difference in change in left hippocampal volume between intervention and control groups (*F*(1,31) =7.13, *p*= 0.012, *ω*^2^ = 0.152). Only the decrease in hippocampal volume observed in control participants significantly differed from zero (*t*(14)= -4.81, *p* <0.001).

Group differences between exercise and control groups, for sub-groups with low (<26) and high (> 26) MoCA scores. Mean (SD) of change are presented for each sub-group.

|  | **MoCA < 26** | | | | | **MoCA ≥ 26** | | | | |  |
| --- | --- | --- | --- | --- | --- | --- | --- | --- | --- | --- | --- |
|  | **Physical activity** | **Control group** | ***F*** | ***p*** | ***ω2*** | **Physical activity** | **Control group** | ***F*** | ***p*** | ***ω2*** | **P value for interaction** |
| ***Change in MRI measures (n)*** | *15* | *14* |  |  |  | *19* | *15* |  |  |  |  |
| Right hippocampal volume (mm^3^) | -282.99 (303.75) | -279.85 (219.65) | 0 | 0.975 | -0.036 | -121.39 (193.62) | -204.02 (139.29) | 1.94 | 0.174 | 0.027 | 0.446 |
| Left hippocampal volume (mm^3^) | -153.72 (258.61) | -223.03 (287.20) | 0.47 | 0.499 | -0.019 | **-57.53 (212.89)** | **-248.77 (200.15)** | **7.13** | **0.012** | **0.152** | 0.319 |
| Global GM volume (mm^3^) | -1824 (20351) | -10223 (13131) | 1.72 | 0.201 | 0.201 | -414.59 (15127) | -13397 (17928) | 5.25 | 0.029 | 0.111 | 0.593 |
| Global WM volume (mm^3^) | -3035 (36524) | 7388 (26183) | 0.77 | 0.388 | 0.388 | -6105 (20862) | 12976 (23489) | 6.28 | 0.018 | 0.134 | 0.530 |
|  |  |  |  |  |  |  |  |  |  |  |  |
| ***Change in cognitive measures (n)*** | *18* | *14* |  |  |  | *22* | *17* |  |  |  |  |
| Two-Back Accuracy^1^ | 0.18 (8.13) | 4.36 (8.81) | 1.66 | 0.209 | 0.023 | 0.57 (7.55) | 8.31 (10.08) | 7.15 | 0.011 | 0.143 | 0.419 |
| Two-Back Reaction Time (ms) | 25.46 (197.49) | 39.16 (189.05) | 0.03 | 0.857 | -0.036 | -22.62 (127.99) | -63.26 (152.59) | 0.78 | 0.385 | -0.006 | 0.520 |
| Flanker (interference)^2^ | -2.9 (14.36) | 2.47 (6.36) | 1.69 | 0.204 | 0.021 | 1.61 (6.71) | -0.18 (13.03) | 0.30 | 0.587 | -0.019 | 0.173 |
| Object Location: identification accuracy (%) | -0.01 (0.07) | 0.03 (0.05) | 2.14 | 0.154 | 0.034 | 0.01 (0.04) | 0.03 (0.03) | 1.38 | 0.247 | 0.01 | 0.477 |
| Object Location: Location errors | -0.17 (2.74) | -0.05 (2.04) | 0.017 | 0.897 | -0.032 | -0.48 (1.28) | -1.31 (1.89) | 2.67 | 0.111 | 0.041 | 0.334 |
| Object Location: Misbinding error | 0.03 (0.12) | 0 (0.15) | 0.52 | 0.475 | -0.015 | 0.04 (0.13) | 0 (0.12) | 0.68 | 0.415 | -0.003 | 0.993 |
| Subjective cognitive complaints (BC-CCI)^3^ | 1.06 (3.72) | -0.43 (4.45) | 1.06 | 0.312 | 0.002 | -1.32 (3.52) | -1.5 (3.76) | 0.02 | 0.879 | -0.026 | 0.485 |

^1^ MOCA < 26: PA_n_ = 17, CG_n_ = 11. MOCA ≥ 26: PA_n_ = 21, CG_n_ = 16.
^2^ MOCA ≥ 26: PA_n_ = 21, CG_n_ = 17.

^3^ MOCA ≥ 26: PA_n_ = 22, CG_n_ = 16.

**Adherence to intervention.**

Based on attendance of the group exercise classes, participants in the intervention arm were divided into high (≥75%; n =23) and low (<75%; n =12) adherence groups. 75% was selected as a cut-off based on the distribution observed in adherence, which ranged from 0-100% (mean = 70.23; median = 77.42%). In a comparison of change across the two adherence groups and control participants, there was no difference in change in MRI or cognitive outcomes.

Differences in change between sub-groups of high adherence (attendance >=75%) and low adherence (<75%) to the exercise intervention. Mean (SD) of change are presented for each sub-group.^1^

|  | **High-adherence group** | **Low-adherence group** | ***F*** | ***p*** | ***ω2*** |
| --- | --- | --- | --- | --- | --- |
| ***Change in MRI measures (n)*** | *20* | *10* |  |  |  |
| Right hippocampal volume (mm^3^) | -195.21 (211.71) | -219.52 (361.13) | 0.05 | 0.817 | 0.033 |
| Left hippocampal volume (mm^3^) | -107.93 (230.16) | -115.06 (254.71) | 0.01 | 0.939 | 0.034 |
| Global GM volume (mm^3^) | -2726 (18069) | 4042 (16973) | 0.972 | 0.333 | 0.192 |
| Global WM volume (mm^3^) | -405.3 (32229) | -10846 (24966) | 0.803 | 0.378 | 0.803 |
|  |  |  |  |  |  |
| ***Change in cognitive measures (n)*** | *23* | *12* |  |  |  |
| Two-Back Accuracy^2^ | 0 (8.23) | 0.33 (7.7) | 0.01 | 0.909 | 0.03 |
| Two-Back Reaction Time (ms) | -8.34 (205.08) | 17.36 (73.33) | 0.17 | 0.679 | 0.025 |
| Flanker (interference)^3^ | -1.49 (13.58) | 2.39 (5.8) | 0.88 | 0.354 | 0.003 |
| Object Location: identification accuracy (%) | -0.01 (0.06) | 0.02 (0.05) | 1.54 | 0.223 | 0.015 |
| Object Location: Location errors | -0.35 (1.81) | -0.53 (2.47) | 0.062 | 0.805 | 0.028 |
| Object Location: Misbinding error | 0.04 (0.12) | 0.02 (0.13) | 0.17 | 0.684 | 0.024 |
| Subjective cognitive complaints (BC-CCI)^4^ | -0.57 (3.74) | 0.75 (4.07) | 0.92 | 0.345 | 0.002 |

^1^ Missing adherence data from 5 participants.
^2^ Two-Back: High_n_ = 22, Low_n_ = 12

^3^ Flanker: High_n_ = 23, Low_n_ = 12

^4^ BCCI: High_n_ = 23, Low_n_ = 12

**Sex.**

Both groups had a higher proportion of female participants (intervention: 65% female; control 55% female). In sub-group analyses by sex, a between group difference in left hippocampal volume change was observed in females (*F*(1,37) =7.76, *p* =0.008, *ω*^2^ =0.148), but not in males (*p* =0.713). Amongst female participants, the decrease in left hippocampal volume was significant in the control group (*t*(15)=-5.84, p <0.001), whereas the change in the intervention group did not differ from zero (*t*(22)=-1.69, p =0.105). Similarly, change in accuracy on the two-back test differed between intervention and control groups in females (*F*(1,37)=13.56, p <0.001, *ω*^2^ =0.244) but not males (p =0.917). T-tests showed that, for females, the increase in accuracy was significant for the control group (*t*(14)= 3.67, *p* = 0.003), but did not differ from zero in the intervention group (*t*(23)= -0.49, *p* =0.63). There were no other differences in change in MRI or cognitive outcomes between groups for either sex.

Differences in change between exercise and control groups in female and male sub-groups. Mean (SD) of change are presented for each sub-group.

|  | **Female** | | | | | **Male** | | | | |  |
| --- | --- | --- | --- | --- | --- | --- | --- | --- | --- | --- | --- |
|  | **Physical activity group** | **Control group** | ***F*** | ***p*** | ***ω2*** | **Physical activity group** | **Control group** | ***F*** | ***p*** | ***ω2*** | **P value for interaction** |
| ***Change in MRI measures (n)*** | *23* | *16* |  |  |  | *11* | *13* |  |  |  |  |
| Right hippocampal volume (mm^3^) | -181.6 (224.45) | -239.31 (157.82) | 0.78 | 0.382 | -0.006 | -215.87 (326.86) | -242.25 (217.33) | 0.06 | 0.816 | -0.04 | 0.795 |
| Left hippocampal volume (mm^3^) | **-85.8 (243.47)** | **-291.27 (199.34)** | **7.76** | **0.008** | **0.148** | -129.57 (226.13) | -168.74 (279.02) | 0.14 | 0.713 | -0.037 | 0.187 |
| Global GM volume (mm^3^) | -4901 (15641) | -16366 (15422) | 5.13 | 0.029 | 0.096 | 7045 (18682) | -6324 (14502) | 3.89 | 0.061 | 0.108 | 0.820 |
| Global WM volume (mm^3^) | 385.87 (28506) | 10813 (21690) | 1.52 | 0.225 | 0.013 | -15491 (26148 | 9621 (28572) | 4.97 | 0.036 | 0.142 | 0.294 |
|  |  |  |  |  |  |  |  |  |  |  |  |
| ***Change in cognitive measures (n)*** | *26* | *17* |  |  |  | *14* | *14* |  |  |  |  |
| Two-Back Accuracy^1^ | **-0.79 (7.94)** | **10.33 (10.91)** | **13.56** | **<0.001** | **0.244** | 2.43 (7.1) | 2.17 (5.15) | 0.01 | 0.917 | -0.04 | **0.009** |
| Two-Back Reaction Time (ms) | 2.43 (193.87) | -7.66 (177.55) | 0.03 | 0.871 | -0.026 | -7.18 (91.25) | -38.88 (172.47) | 0.36 | 0.555 | -0.025 | 0.804 |
| Flanker (interference)^2^ | -1.06 (13.17) | 0.95 (12) | 0.26 | 0.616 | -0.018 | 0.71 (4.46) | 1.1 (8.72) | 0.02 | 0.888 | -0.038 | 0.765 |
| Object Location: identification accuracy (%) | 0.62 (6.31) | 3.21 (3.99) | 2.26 | 0.14 | 0.029 | 0.36 (3.93) | 2.2 (4.54) | 1.39 | 0.249 | 0.014 | 0.779 |
| Object Location: Location errors | -0.16 (2.41) | -0.79 (2.07) | 0.79 | 0.379 | -0.005 | -0.68 (1.09) | -0.68 (2.05) | <0.001 | 0.992 | -0.037 | 0.536 |
| Object Location: Misbinding error | 0.03 (0.13) | 0 (0.14) | 0.44 | 0.509 | -0.013 | 0.05 (0.12) | 0.02 (0.12) | 0.99 | 0.327 | 0 | 0.766 |
| Subjective cognitive complaints (BC-CCI)^3^ | -0.38 (3.6) | -0.59 (3.47) | 0.03 | 0.855 | -0.023 | 0 (4.17) | -1.54 (4.82) | 0.79 | 0.383 | -0.008 | 0.496 |

^1^ Female: PA_n_ = 24, CG_n_ = 15. Male: PA_n_ = 14, CG_n_ = 12.

^2^ Female: Male: PA_n_ = 13, CG_n_ = 14.

^3^ Female: Male: PA_n_ = 14, CG_n_ = 13

**Appendix F. Partial correlations between change in left hippocampal volume and cognitive outcomes in the physical activity group, adjusting for age, sex and education.**

|  | **Partial R** | **P-value** |
| --- | --- | --- |
| ***Changes in cognitive measures*** |  |  |
| 2Back Accuracy | 0.058 | 0.763 |
| 2Back Reaction Time | 0.153 | 0.418 |
| Flanker (interference) | 0.016 | 0.932 |
| Object Location: identification accuracy (%) | 0.267 | 0.147 |
| Object Location: Location errors | 0.073 | 0.697 |
| Object Location: Misbinding error | 0.11 | 0.555 |
| Subjective cognitive complaints (BC-CCI) | -0.09 | 0.619 |

**Appendix G. Partial correlations between change in SPPB and MRI and cognitive outcomes in the physical activity group, adjusting for age, sex and education.**

|  | **Partial R** | **P-value** |
| --- | --- | --- |
| ***Changes in MRI outcomes*** |  |  |
| Left Hippocampal volume (mm^3^) | 0.047 | 0.808 |
| Right Hippocampal volume (mm^3^) | 0.128 | 0.509 |
| ***Changes in Cognitive outcomes*** |  |  |
| 2Back Accuracy | 0.454 | 0.008 |
| 2Back Reaction Time | 0.019 | 0.915 |
| Flanker (interference) | 0.019 | 0.915 |
| Object Location: identification accuracy (%) | 0.098 | 0.582 |
| Object Location: Location errors | -0.135 | 0.447 |
| Object Location: Misbinding error | 0.085 | 0.635 |
| Subjective cognitive complaints (BC-CCI) | -0.373 | 0.030 |

**Appendix H. Mixed ANOVA of group effect on left hippocampal volume.**


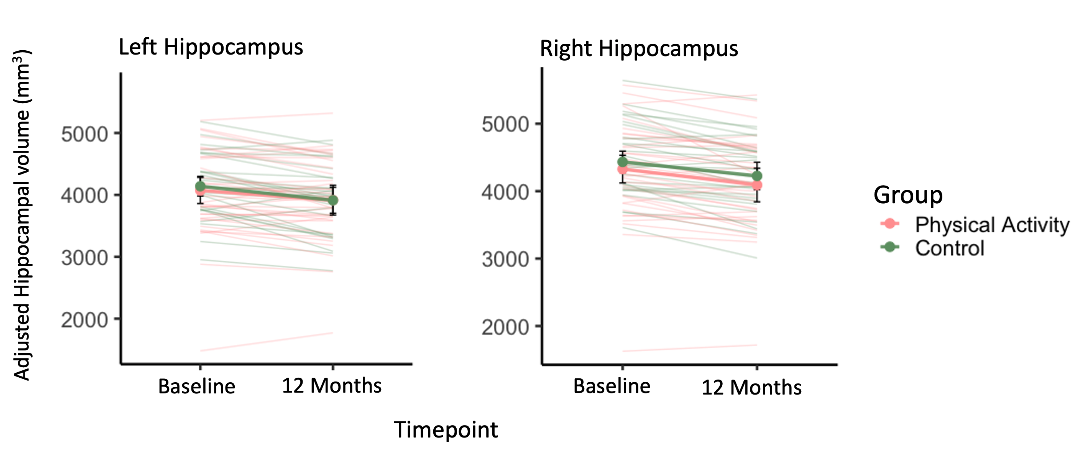


In order to ensure that the effect on left hippocampal volume was not driven by (non-significant) baseline differences in hippocampal volume, a mixed 2x2 ANOVA with group (physical activity and control) as a between-group factor and time (0 and 12 months) as a within-subjects factor was also conducted. The analysis revealed a significant interaction between time and group (F(1,61) = 5.12, p = 0.027, η^2^= 0.003). Individuals in the physical activity group showed reduced decline in left hippocampal volume than individuals in the control group. This was in line with our results from the one-way ANOVA on change in left hippocampal volume.

**Appendix I. Mean (SD) of outcome measures at 6 and 12 months are presented for each group.**

|  | **6 Months** | | **12 Months** | |
| --- | --- | --- | --- | --- |
|  | **Physical activity** | **Control group** | **Physical activity** | **Control group** |
| ***MRI measures (n)*** | *39* | *33* | *34* | *29* |
| Right hippocampal volume (mm^3^)* | 4256.93 (855.60) | 4426.06 (533.39) | 4091.19 (713.89) | 4225.73 (535.35) |
| Left hippocampal volume (mm^3^)* | 4046.77 (819.18) | 4160.0 (520.74) | 3915.6 (688.74) | 3913.12 (550.03) |
|  |  |  |  |  |
| ***Cognitive measures (n)*** | *43* | *35* | *40* | *30* |
| 2Back Accuracy | 29.51 (9.79) | 27.5 (9.92) | 30.23 (8.52) | 32.38 (7.51) |
| 2Back Reaction Time | 1226.39 (203.1) | 1236.45 (191.68) | 1274.95 (195.62) | 1217.76 (230.83) |
| Flanker (interference) | 12.70 (7.86) | 13.2 (6.47) | 12.32 (8.96) | 15.80 (6.97) |
| Object Location: identification accuracy (%) | 88.3 (5.3) | 87.4 (5.9) | 88.91 (6.5) | 89.26 (6.94) |
| Object Location: Location errors | 8.83 (4.53) | 8.62 (8.62) | 7.66 (2.75) | 8.38 (2.29) |
| Object Location: Misbinding error | 0.121 (0.086) | 0.131 (0.084) | 0.163 (0.072) | 0.184 (0.075) |
| Subjective cognitive complaints (BC-CCI) | 6.23 (3.60) | 6.57 (3.17) | 6.23 (3.53) | 5.8 (3.72) |

*Hippocampal volumes were for adjusted for head size using the scaling factor from SIENAX.
